# Estimation of free-roaming dog populations using Google Street View: A methodological study

**DOI:** 10.1371/journal.pone.0305154

**Published:** 2025-07-31

**Authors:** Guillermo Porras, Elvis W. Diaz, Micaela De la Puente-León, Cesar M. Gavidia, Ricardo Castillo-Neyra

**Affiliations:** 1 Zoonotic Disease Research Lab, One Health Unit, School of Public Health and Administration, Universidad Peruana Cayetano Heredia, Lima, Peru; 2 Facultad de Medicina Veterinaria, Universidad Nacional Mayor de San Marcos, Lima, Perú; 3 Department of Biostatistics, Epidemiology & Informatics, Perelman School of Medicine at University of Pennsylvania, Philadelphia, Pennsylvania, United States of America; 4 Department of Pathobiology, School of Veterinary Medicine at University of Pennsylvania, Philadelphia, Pennsylvania, United States of America; UFSJ: Universidade Federal de Sao Joao del-Rei, BRAZIL

## Abstract

Controlling and eliminating zoonotic pathogens such as rabies virus, *Echinococcus granulosus*, and *Leishmania spp*. require quantitative knowledge of dog populations. Dog population estimates are fundamental for planning, implementing, and evaluating public health programs. However, dog population estimation is time-consuming, requires many field personnel, may be inaccurate and unreliable, and is not without danger. Our objective was to evaluate a remote method for estimating the population of free-roaming dogs using Google Street View (GSV). Adopting a citizen science approach, participants from Arequipa and other regions in Peru were recruited using social media and trained to use GSV to identify and count free-roaming dogs in 20 urban and 6 periurban communities. We used correlation metrics and negative binomial models to compare the counts of dogs identified in the GSV imagery with accurate counts of free-roaming owned dogs estimated via door-to-door (D2D) survey conducted in 2016. Citizen scientists detected 862 dogs using GSV. After adjusting by the proportion of streets that were scanned with GSV we estimated 1,022 free-roaming dogs, while the 2016 D2D survey estimated 1,536 owned free-roaming dogs across those 26 communities. We detected a strong positive correlation between the number of dogs detected by the two methods in the urban communities (r = 0.85; p < 0.001) and a weak correlation in periurban areas (r = 0.36; p = 0.478). Our multivariable model indicated that for each additional free-roaming dog estimated using GSV, the expected number of owned free-roaming dogs decreased by 2% in urban areas (p < 0.001) and increased by 2% in peri-urban areas (p = 0.004). The type of community (urban vs periurban) had an effect on the predictions, and fitting the models in periurban communities was difficult because of the sparsity of high-resolution GSV images. Using GSV imagery for estimating dog populations is a promising tool, especially in urban areas. Citizen scientists can help to generate information for disease control programs in places with insufficient resources.

## Introduction

Free-roaming dogs are a public health problem because of the transmission of pathogens that affect both animals [1,2] and people [3,4], as well as through bites that can cause disfiguration [5] and psychological harm [6]. Dog bites are a particular serious health issue in Arequipa, Peru, where up to 14% of adults experience dog bites annually [7]. The global dog population has been estimated at more than 990 million, according to the Wildlife Conservation Society (WCS), of which approximately 700 million are free-roaming dogs [8]. The free-roaming population is comprised of owned dogs that are allowed outside of houses without restriction, previously owned dogs that escaped, were abandoned, or got lost, and dogs born ownerless. For rabies, knowledge of the free-roaming dog population is crucial since these dogs have higher contact rates and may be responsible for most of the transmission and persistence of the rabies virus [9]. Detailed information on these dog subpopulations is crucial for programs aimed at eliminating dog-mediated diseases [10]. However, planning, implementation, and evaluation of current control strategies are hampered by a lack of information on free-roaming dog population size [11].

Methods recommended by the World Health Organization (WHO) for estimating free-roaming dog populations include total or direct counts, estimation method by catch rates, method of recapture, and the Beck or photographic recapture method [12]. Other reported methods include mark-resight procedures, simple dog counts with encounter rates, monitoring dogs marked during vaccination through home visits, and the Pasteur method that involves surveys and direct counts [13]. However, these methods can result in variable estimates in free-roaming dog population [13,14], are time-consuming (e.g., direct counts) [14], and require trained personnel [15,16]. In addition, many of these surveys can be expensive [17]. Program costs are particularly concerning in resource-constrained settings, where inadequate resources are assigned to public health strategies responsible for managing and preventing the spread of dog-mediated diseases. Hence, there is a need to develop new and more cost-effective methods to estimate free-roaming dog populations.

One potential alternative is to use an already existing and easily accessible database like the images captured by Google Street View (GSV). GSV is made up of billions of images to display a virtual representation of our world on Google Maps and Google Earth, allowing users to explore the world virtually and its content comes from Google and its partners [18]. This technology provides virtual panoramas using an advanced camera system mounted on moving vehicles; these vehicles revisit areas to update the imagery [19]. The available up-to-date imagery and the ease of use of GSV may facilitate its application in digital epidemiology studies [20–22]. GSV imagery has been successfully used, for example, to analyze the distribution of moths in trees for ecological research [23], and assess the nesting habitat of vultures for conservation purposes [24]. However, its potential to estimate the free-roaming dog population has not yet been explored.

Our aim was to evaluate a remote methodology for estimating the free-roaming dog population using GSV imagery. We then compared these results with previous data gathered by a 2016 D2D survey. Developing a remote method to estimate the free-roaming dog population could be useful in countries where the dog population size is unknown and other methods are unfeasible [16].

## Methods

In this methodological study, we compared counts of free-roaming dogs identified through GSV imagery with those obtained from D2D surveys conducted in 2016 in the same study communities. Volunteers (citizen scientists) reviewed GSV imagery—captured around or before 2016—to detect and count free-roaming dogs. We then used correlation metrics and regression models to assess the agreement between the two data sources.

### Study area

**Fig 1:**
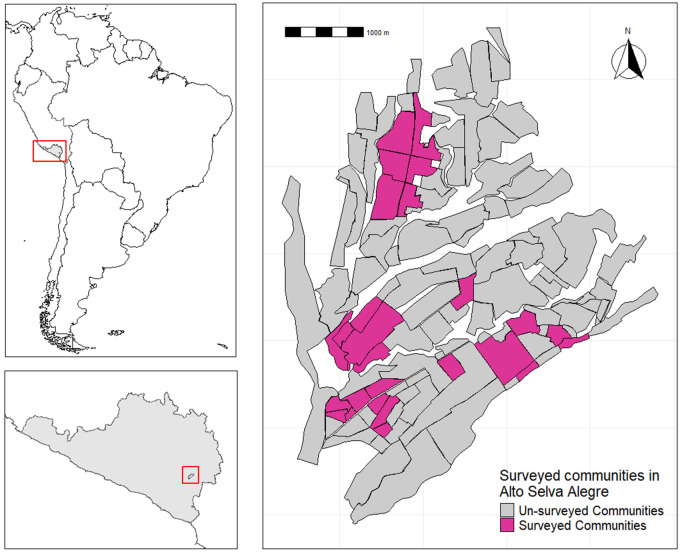
Location of communities included within the study, Alto Selva Alegre, Arequipa, Peru.

The study was undertaken in Arequipa City, southern Peru, where dog rabies has re-emerged as a worrying threat since 2015 [25]. Specifically, this study was conducted in the district of Alto Selva Alegre (ASA) (human population for 2016: 83,310). A total of 26 communities (administrative divisions within a district) were included in the study, 20 in urban areas and 6 in periurban areas (Fig 1).

### Citizen scientists’ recruitment and training on GSV imagery search

We recruited twenty-two citizen scientists through Facebook pages related to veterinary medicine, veterinary schools, and animal welfare. Additional participants from the veterinary school at Universidad Nacional Mayor de San Marcos were invited via WhatsApp. All participants received: i) a digital manual with the search protocol; ii) an Excel data collection sheet; iii) a Google Maps link to their assigned area (polygon); and iv) the GSV image capture date. The search protocol followed the World Society for the Protection of Animals (WSPA) protocol [26]. Citizen scientists were instructed to analyze GSV imagery captured in September 2013—the most widely available date—to identify free-roaming dogs. WhatsApp was used to provide remote support during the COVID-19 pandemic restrictions.

### Dog detection in GSV imagery

To refine the search protocol and assess the reliability of citizen scientists’ observations, we conducted a pilot study in six communities of Arequipa. To assess the optimal number of observers per area, we randomly assigned four citizen scientists to each of six pilot communities. Each citizen scientist independently scanned the same area, blinded to the results of their peers. A proficient researcher (GP), serving as a gold standard, also reviewed these six communities. It is possible that detectability increases with the number of observers per area. Therefore, we compared the number of unique dogs detected by one, two, three, and four citizen scientists to the count obtained by the proficient researcher. On average, one citizen scientist detected 70.7% of the free-roaming dogs identified by the proficient researcher; this increased to 88.7% with two observers, 95.7% with three, and 100% with four. Based on these findings, we defined four citizen scientists as the necessary number for accurate dog counts using GSV in subsequent analyses.

We selected 26 communities for which both 2016 D2D survey data and GSV imagery were available to compare free-roaming dog counts. These 26 communities were assigned to the 22 citizen scientists, ensuring each community had a different combination of four citizen scientists. The search involved scanning GSV imagery in virtual tours. At each stopping point (approximately 7 meters apart), the citizen scientists turned 360º to identify and record free-roaming dogs. They registered unique sightings in the Excel sheet along with: i) the month and year (2013) when Google captured the image; ii) the community name; and iii) the image link. Only dogs walking freely were included. The data were then emailed to the proficient researcher for evaluation. GSV imagery is not available for all roads and streets. To adjust for this, we estimated the exact proportion of roads and streets with available GSV imagery in each community and corrected the observed counts of free-roaming dogs accordingly to obtain community-level estimates.

The proficient researcher verified the data collected reviewing each image link in the Excel database. This process involved confirming that dogs were free-roaming, located within the target area, not double-counted, and associated with the correct image date. Each dog was systematically coded and georeferenced to enable comparison across the combination of four citizen scientists in a given community. The maximum number of detectable free-roaming dogs in that area by four citizen scientists was considered the total discoverable number of dogs in each community by GSV.

### Door-to-door household survey

In 2016, the Zoonotic Disease Research Laboratory of the Universidad Peruana Cayetano Heredia conducted a D2D survey in 42 communities (21 urban and 21 periurban) within the district of ASA. These communities are discrete, well-recognized sub-district areas with typically 500–1000 houses. The D2D survey occurred between June 1st and August 7th, 2016. Each household in the study communities was invited to participate in the survey. Closed and hesitant households were visited up to three times to reduce selection bias. An adult in each participant household provided informed consent and then responded verbally to the structured survey, which took approximately 15 minutes. The D2D survey was based on the rabies literature and our qualitative studies of local communities [27]. Data were collected on the household’s dogs, including number of dogs, sex, age, level of restriction, data on household characteristics, and dog owner’s demographics, aiming to characterize the dog population. All houses in the study communities were geocoded and survey data were linked to household coordinates. These surveys helped to estimate the total owned dog population and the owned free-roaming dog population in every community.

### Statistical analysis

**Fig 2:**
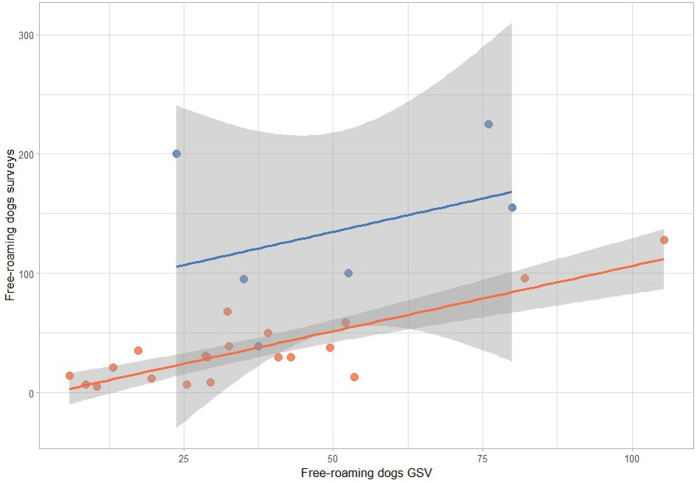
Free-roaming dogs counts from 2013 Google Street View (GSV) imagery versus 2016 door-to-door (D2D) survey in urban (red) and periurban (blue) communities. Regression lines with 95% confidence bands are shown.

We employed Pearson’s coefficient to assess the correlation between the number of free-roaming dogs identified using GSV and of those with free access to the street as reported in the 2016 D2D survey. We explored different model distributions for the number of free-roaming dogs, including Poisson, negative binomial, and normal distributions. The distribution of the number of free-roaming dogs from the 2016 D2D survey was analyzed using the ‘Fitdistrplus’ package in R, which allowed us to fit different statistical distributions and determine the best-fitting parameters. Additional data from Google Maps and the 2016 D2D survey that were explored as covariates were i) The length of streets scanned using GSV (search distance); ii) community area; iii) number of houses; and iv) community classification (urban or periurban). To estimate confidence bands around our regression lines (Fig 2) we used the geom_smooth function from the R package ‘ggplot’.

Based on the distribution of the response variable (free-roaming dog count based on a D2D survey), we selected a negative binomial regression model to estimate the association between the predictor variables (dogs detected in 2013 GSV imagery, search distance, community area, number of houses), and the response variable. Model building was conducted using a backward stepwise regression approach, and multicollinearity was assessed by calculating the degree of correlation between the potential predictor variable and the variance inflation factor (VIF). A VIF value higher than 10 was used to determine multicollinearity among the regression variables.

For model selection, we used Akaike’s Information Criteria (AIC) and the statistical significance of the variables included in our model. For the final model, we used a negative binomial regression model with the number of dogs detected via GSV and number of houses as predictors, and community type (urban vs. peri-urban) as an effect modifier. All analyses and figures were produced in R [28], with a statistical significance level of 0.05.

### Ethics statement

Ethical approval for the 2016 D2D survey was obtained from Universidad Peruana Cayetano Heredia (approval number: 65369), Tulane University (approval number: 14–606720), and the University of Pennsylvania (approval number: 823736). All human subjects in this study were adults and provided written informed consent. We did not assign any animals to experimental interventions, did not confine any animals, and did not touch any animals.

## Results

Using 2013 GSV imagery, citizen scientists identified 862 unique free-roaming dogs across 26 communities (20 urban and 6 periuban), covering a combined area of 223.3 hectares and scanning 59.38 kilometers of streets and roads. GSV imagery was available for 73.2% of roads and streets in periurban communities (range: 56.0–88.5%), compared to 86.5% in urban communities (range: 52.5–100%). After adjusting for image coverage, the estimated number of dogs across the 26 communities was 1,022. The average number of free-roaming dogs estimated (after adjustment) per periurban community was 50.8 and 35.9 per urban community, but this difference was not statistically significant (Table 1). We identified and removed 15 dogs that were counted at least twice (double-counting rate = 1.74%).

**Table 1:** Free-roaming dogs counted with 2013 GSV imagery and estimated with the 2016 D2D survey in study urban and periurban communities in Arequipa, Peru.

Variable	Periurban communities (n = 6)	Urban communities (n = 20)	p-value^*^
Free-roaming dogs estimated from the 2016 D2D survey – mean (range)	135.7 (39–225)	36.1 (5–28)	<0.001
Number of dogs found in GSV without adjustment^**^ - mean (range)	38 (16–64)	31.7 (4–89)	0.429
Free-roaming dogs estimated with GSV with adjustment^**^ - mean (range)	50.8 (23.8- 79.9)	35.9 (5.84–105)	0.176
Total length of streets in kilometers – mean (range)	2.2 (1.3–3.6)	2.3 (0.4–7.2)	0.605
Total study area – Ha – mean (range)	13 (7.7–17.2)	7.4 (1.3–27.4)	0.007
Number of houses – mean (range)	287.7 (143–501)	219.2 (31 −735)	0.139

*Estimated with Wilcoxon test;

**adjusted by the proportion of roads/streets with available GSV imagery

A single citizen scientist found an average of 70.68% free-roaming dogs compared to the total dogs detected by the proficient researcher in a community. That proportion increased to 88.7% for two citizen scientists and 95.67% for three citizen scientists. Finally, four citizen scientists were able to detect 100% of the free-roaming dogs, so we established this as the optimal number of searchers to accurately determine the number of free-roaming dogs using GSV. In instances, when four citizen scientists in the same community detected more free-roaming dogs compared to the proficient researcher, that particular count was designated as the gold standard for the respective community. Only in four communities a set of 3 or 4 citizen scientists found more dogs than the proficient researcher.

A total of 4370 households were surveyed door-to-door, with 563 in periurban communities (n = 6) and 3807 in urban communities (n = 20). Survey participation rates were 61.6% in periurban areas and 88.8% in urban areas. The total number of dogs in these communities was adjusted by the participation rate per community. With the 2016 D2D survey, we estimated a total dog population of 4714 and a subpopulation of owned free-roaming dogs of 1536 (Table 1). Also, with the 2016 D2D survey, the dog population density in the periurban areas was 542 dogs/km2 while in urban areas was 3093 dogs/km2.

We found strong correlations between the response variable and all the predictor variables in urban areas (Table 2) and variable correlations, ranging from weak to strong in periurban areas (Table 2). Specifically, free-roaming dogs detected by 2013 GSV imagery and owned free-roaming dogs estimated with the 2016 D2D survey showed a strong and significant correlation in urban areas (r = 0.85; p < 0.001), and a weak and not significant correlation in periurban areas (r = 0.36; p = 0.478) (Table 2).

**Table 2:** Pearson correlation coefficients between the number of free-roaming dogs estimated with the 2016 D2D survey and the counts of dogs identified through 2013 GSV imagery in urban (red) and periurban (blue) communities in Arequipa, Peru.

Pearson Correlation						
**Variables**	**Dogs found in GSV**	**Free-roaming dogs from 2016 D2D surveys**	**Total dogs from 2016 D2D surveys**	**Scanned roads in km**	**Area (Ha) of the community**	**Number of houses in the community**
**Dogs found in GSV**	**–**	**0.85** ^**^	**0.89** ^**^	**0.88** ^**^	**0.83** ^**^	**0.88** ^**^
**Free-roaming dogs from 2016 D2D surveys**	**0.36**	**–**	**0.88** ^**^	**0.85** ^**^	**0.87** ^**^	**0.8** ^**^
**Total dogs from 2016 D2D surveys**	**0.39**	**0.99** ^**^	**–**	**0.98** ^**^	**0.95** ^**^	**0.98** ^**^
**Scanned roads in km**	**0.78**	**0.8**	**0.78**	**–**	**0.96** ^**^	**0.97** ^**^
**Area (Ha) of the community**	**0.1**	**0.27**	**0.28**	**0.24**	**–**	**0.92** ^**^
**Number of houses in the community**	**0.17**	**0.89** ^*^	**0.82** ^*^	**0.68**	**0.35**	**–**

*Correlation coefficient with a p-value <0.05;

**Correlation coefficient with a p-value <0.01

In both, urban and periurban communities, there was a clear positive and almost linear association between the number of free-roaming dogs detected with 2013 GSV imagery and the number of free-roaming dogs reported in the 2016 D2D survey (Fig 2). However, the confidence bands around the periurban regression line were substantially wider compared to those around the urban regression line.

In urban areas, the univariable negative binomial models showed a highly significant statistical positive association between the number of free-roaming dogs reported in the 2016 D2D survey and several variables, including the number of dogs detected with 2013 GSV imagery, the number of houses in the community, the total length of roads, and the area of the community (p < 0.001; Table 3A). In contrast, in periurban areas, the number of dogs detected with 2013 GSV imagery was not statistically associated with the number of free-roaming dogs reported in the 2016 D2D. The final multivariable model for both urban and periurban communities included the number of dogs detected with 2013 GSV imagery, the number of houses, and the type of community (urban or periurban) as a modifier of the association between the response variable and the number dogs detected with 2013 GSV imagery. To improve interpretability, the multivariable models were stratified by type of community and presented separately ("Tables 3A and B). Our multivariable model indicated that for each additional free-roaming dog estimated using GSV, the expected number of owned free-roaming dogs decreased by 2% in urban areas (p < 0.001) and increased by 2% in peri-urban areas (p = 0.004). This interpretation indicates an exponential association between GSV-estimated free-roaming dogs and owned free-roaming dogs, consistent with the multiplicative nature of count models such as negative binomial regression. While the slopes in Fig 2 look quite similar, the coefficients indicate otherwise. A marked difference in Fig 2 are the intercepts between urban and periurban areas. In urban areas, zero dogs detected via GSV equates zero dogs estimated with D2D survey; however, in periurban areas, the intercept is close to 70, suggesting that many free-roaming dogs reported in the D2D survey are underestimated by the GSV method. The confidence bands in Fig 2 also reflect the model’s differing predictive ability based on community.

**Table 3:** A. Association coefficients for free-roaming dogs using Google Street View in urban communities of Arequipa, Peru. B. Association coefficients for free-roaming dogs using Google Street View in periurban communities of Arequipa, Peru.

A				
Variable	Univariable model, rate ratio (95% CI)	p-value	Multivariable model, rate ratio (95% CI)	p-value
Dogs found using GSV	1.02 (1.01–1.04)	<0.001	0.98 (0.97–0.99)	<0.001
Number of houses	1.00 (1.00–1.01)	<0.001	1.00 (1.00–1.01)	0.001
Distance traveled (Km)	1.33 (1.16–1.55)	<0.001	–	–
Area (Ha)	1.08 (1.04–1.13)	<0.001	–	
**B**				
Variable	Univariable model, rate ratio (95% CI)	p-value	Multivariable model, rate ratio (95% CI)	p-value
Dogs found using GSV	1.01 (0.99–1.03)	0.381	1.02 (1.01–1.04)	0.004
Number of houses	1.00 (1.00–1.01)	0.001	1.00 (1.00–1.01)	0.001
Distance traveled (Km)	1.68 (1.12–2.60)	0.007	–	–
Area (Ha)	1.04 (0.92–1.16)	0.468	–	–

The estimated equations to calculate the predicted value of dogs in the 2016 D2D survey using the variables, Houses, *Dogs* found in GSV, and the interaction between variables Dogs found in GSV and Type of Community Urban are the following:

Y = Dogs with free access to the street reported in the 2016 D2D survey

Y ~ Negative Binomial (λ)


$$\begin{array}{cc} log(\lambda )\; & =\;\beta_{0}\;+\;\beta_{1}\;*\;(\#\;Dogs\;found\;in\;GSV)\;+\;\beta_{2}\;*\;(\#\;Houses)\;\\ & \;+\;\beta_{3}\;*\;(\#\;Dogs\;found\;in\;GSV)*(Type\;of\;Community\;Urban)\;\\ & \;+\;\beta_{4}\;*\;(Type\;of\;Community\;Urban) \end{array}$$

## Discussion

The results of this study in 26 urban and periurban communities of Arequipa, Peru, showed a strong correlation between the population of free-roaming dogs reported in the 2016 D2D survey versus free-roaming dogs detected using 2013 GSV imagery. The prediction model with the best fit included the number of dogs detected with GSV, the number of households, and the interaction with the type of community (urban or periurban), highlighting the heterogeneous ecology of free-roaming dogs within cities and evidencing that this new method is viable, at least in well-urbanized areas [29,30]. In periurban communities, predictability was reduced, maybe because the sample size in periurban areas was relatively small and there was high dispersion of observed values. Additionally, the virtual exploration of periurban communities using GSV images was less comprehensive relative to urban areas due to the unavailability of GSV images in certain areas or streets. This observed sparsity may be caused by the challenging periurban terrain that impedes the Google vehicles from traveling some streets to capture images, an issue that has been reported by others [31]. However, it is also possible that the ecology of free-roaming dogs is very different in periurban areas. Urban and periurban areas differ in many aspects that impact the epidemiology of rabies and other infectious diseases, including topography, access to health services and other public goods, socioeconomic status and animal and human demography [7,32–34]. Differences in these factors could impact the ecology of free-roaming dogs.

The number of citizen scientists reviewing GSV imagery was key to accurately counting free-roaming dogs. Four trained observers consistently detected nearly 100% of the dogs identified by the proficient researcher, a finding aligned with previous studies using GSV to located wildlife, such as vulture nests [23]. Lower detection rates with fewer observers may reflect variability in search behavior, visual acuity, attention, or motivation—factors noted in other studies [35,36].

When comparing the number of dogs detected with 2013 GSV imagery with those reported in the 2016 D2D survey, the results varied among communities. At the community level —a small geographic unit—GSV both overestimated and underestimated free-roaming dog populations in some areas; however, the overall prediction of urban free-roaming dogs based on survey data was accurate. Other authors have noted difficulties when comparing alternative methods to survey; for example, counting dogs with drones underestimates the total dog population because dogs may hide under trees or cars, avoiding detection by the drones [35]. On the contrary, we observed that GSV, generally, allows detection of dogs under trees and even cars. A potential challenge when observing and recording free-roaming dogs is the risk of double-counting. In Tulum, Mexico, researchers using a modified photographic capture-recapture method reported a double counting rate of 0.32% [37]. In our study, 1.74% of the detected dogs were counted more than once. The Tulum study also noted that their method may underestimate the dog population, as transects were conducted on only a subset of streets [37]. In contrast, our study covered all streets within each study community.

Other methods involving direct observation have been evaluated. In Ecuador, Cardenas et al. compared two methods of estimation of free-roaming dogs on transects (capture-recapture and distance sampling) [16]. They detected twice as many dogs using the distance sampling method compared to the capture-recapture method in urban areas. They also found that low light at the time of surveys affected the accuracy of individual identification and errors in calculating the distance between the animal and the observer caused underestimation of the density of dogs/km² in rural areas. Compared to those methods, counting dogs with GSV requires less time and logistical material. We found the GSV performance was better in urban areas compared to periurban areas. Similarly, Cardenas et al. proposed alternative methods for different areas or even combining different methods to increase accuracy [16]. Estimating the dog population in Arequipa, as in many parts of Latin America, remains a major challenge [15]. Traditional approaches relying on fixed human-to-dog ratios overlook spatial variability [32]and introduce significant uncertainty. This has hampered efforts to assess and improve mass dog vaccination campaigns. Innovative tools like GSV can help reduce this uncertainty and support more targeted disease control strategies [38].

The method employed in our study showed some limitations. Firstly, the variability of images in GSV due to periodic updates may affect the consistency of dog observations made by citizen scientists. Additionally, the availability of the Street View function in certain areas (e.g., alleys, unpaved secondary roads) may limit the search for free-roaming dogs. Furthermore, uncertainties regarding the timing and frequency of image capture introduce unknown intervals for analysis. It is important to recognize that our estimation of free-roaming dogs using GSV includes unowned dogs, whereas the 2016 D2D survey exclusively recorded owned dogs reported by owners [39]. Additionally, in GSV images, ownership status cannot be reliably inferred based on the presence of a collar, as many owned dogs do not wear collars, while some community-cared but unowned dogs may. Importantly, the Peruvian Ministry of Health estimates that 90% of free-roaming dogs in urban areas are owned [40], suggesting that the potential influence of unaccounted stray dogs on our results may be limited. Nevertheless, estimating the stray dog population remains essential to provide a comprehensive understanding of the target population for zoonotic disease control programs. Despite these limitations, utilizing GSV presents advantages over traditional methodologies, particularly during circumstances like the COVID-19 pandemic or in resource-limited settings, as it eliminates the need for field trips by trained personnel [11,14,40,41]. Moreover, the periodic updating of the Google Street View database [19] enhances the potential for timely updates on free-roaming dog populations across different regions.

The present study demonstrates Google Street View can be a useful tool for obtaining accurate and reliable estimates of the free-roaming dog population. The method also offers several advantages over the 2016 D2D survey, transect counts, or photographic capture-recapture. GSV is already used for construction projects, geological evaluations, ecological studies, and even assessment of chronic diseases [23,24,41,42]. Pairing GSV with newer technologies such as machine learning or artificial intelligence could enhance its potential and make it faster and more available for programs without human resources to conduct virtual searches. In addition to obtaining the dog population size, with GSV it is also possible to obtain the exact geographic coordinates of dogs, which facilitates the analysis of their distribution relative to biological events and geographical features. Information from such analysis could feed control programs for various dog-mediated diseases of public health concern [3,43] as well as diseases threatening other animal species [44,45]. The use of GSV imagery for dog population estimation provides an economically viable approach to address the limited knowledge about free-roaming dogs in urbanized areas. The method requires minimal resources and has the potential to inform effective public health policies and programs addressing free-roaming dogs.
